# ATP Release from Human Airway Epithelial Cells Exposed to *Staphylococcus aureus* Alpha-Toxin

**DOI:** 10.3390/toxins8120365

**Published:** 2016-12-06

**Authors:** Romina Baaske, Mandy Richter, Nils Möller, Sabine Ziesemer, Ina Eiffler, Christian Müller, Jan-Peter Hildebrandt

**Affiliations:** Animal Physiology and Biochemistry, Ernst Moritz Arndt-University, Felix Hausdorff-Strasse 1, B.10.06, D-17489 Greifswald, Germany; RominaBaaske@hotmail.de (R.B.); mandy.richter91@t-online.de (M.R.); nils.moeller@uni-greifswald.de (N.M.); sabine.ziesemer@uni-greifswald.de (S.Z.); ina.eiffler@uni-greifswald.de (I.E.); christian.mueller@uni-greifswald.de (C.M.)

**Keywords:** ATP efflux, airway epithelial cells, *Staphylococcus aureus*, virulence factor, alpha-toxin

## Abstract

Airway epithelial cells reduce cytosolic ATP content in response to treatment with *S. aureus* alpha-toxin (hemolysin A, Hla). This study was undertaken to investigate whether this is due to attenuated ATP generation or to release of ATP from the cytosol and extracellular ATP degradation by ecto-enzymes. Exposure of cells to rHla did result in mitochondrial calcium uptake and a moderate decline in mitochondrial membrane potential, indicating that ATP regeneration may have been attenuated. In addition, ATP may have left the cells through transmembrane pores formed by the toxin or through endogenous release channels (e.g., pannexins) activated by cellular stress imposed on the cells by toxin exposure. Exposure of cells to an alpha-toxin mutant (H35L), which attaches to the host cell membrane but does not form transmembrane pores, did not induce ATP release from the cells. The Hla-mediated ATP-release was completely blocked by IB201, a cyclodextrin-inhibitor of the alpha-toxin pore, but was not at all affected by inhibitors of pannexin channels. These results indicate that, while exposure of cells to rHla may somewhat reduce ATP production and cellular ATP content, a portion of the remaining ATP is released to the extracellular space and degraded by ecto-enzymes. The release of ATP from the cells may occur directly through the transmembrane pores formed by alpha-toxin.

## 1. Introduction

Many strains of *Staphylococcus aureus* produce virulence factors that are secreted to the external medium and mediate pathogenicity [1,2]. One of the major virulence factors generated by pathogenic *S. aureus* strains is the pore-forming alpha-toxin (hemolysin A, Hla) [3,4]. Expression of the *hla* gene is induced in bacterial cultures when bacteria reach critical densities [5]. Hla is secreted from the cells as a 33 kDa monomer that attaches to the outer surface of eukaryotic host cells. At low concentrations, Hla may associate with eukaryotic plasma membranes in a specific manner with half-maximal binding at 1–2 nmol/L (30–60 ng/mL) [6]. The metalloproteinase ADAM10 may be an important docking site for Hla [7]. Areas of specific lipid composition may also be involved in Hla attachment to the host cell plasma membrane [8]. Non-specific binding, however, occurs at concentrations at >1 μmol/L (33 μg/mL) [6], which may result in cell disruption (e.g., hemolysis) by disturbing the lipid barrier. 

Upon specific binding to the eukaryotic plasma membrane, Hla monomers form ring-like heptameric structures (pre-pores) before all subunits of such a complex undergo a simultaneous conformational change that inserts the hairpins of each of the subunits deeply into the plasma membrane of the host cell [9]. This results in the formation of a barrel-shaped transmembrane pore, which provides an open connection between cytosol and extracellular space with an inner diameter of approximately 1.4 nm at its narrowest site [1,10]. The effective diameter of the pore, however, may be even smaller as indicator molecules such as acridine orange (265 g/mol), 2',7'-bis-(2-carboxyethyl)-5-(and-6)-carboxyfluorescein (BCECF) (approx. 600 g/mol) or Indo-1 (approx. 500 g/mol) do not leave the cells upon treatment with sub-lethal concentrations of hemolysin A [11,12]. On the other hand, Hla pores inserted in artificial membranes may even conduct DNA molecules that have been tested as a means for developing new sequencing techniques [13]. Moreover, studies using various cell types have shown that Hla-treatment results in a decline in intracellular ATP concentration [11,14,15,16,17], which has been interpreted as an indication that the Hla-pore itself may be permeable for ATP as originally suggested by Bhakdi and Tranum-Jensen [3]. However, some (esp. epithelial) cells release ATP as a physiological response to stressful stimuli [18]. While there is now clear evidence that the cystic fibrosis transmembrane conductance regulator (CFTR), which had been previously implicated with release of ATP from airway epithelial cells [19], does not provide an exit pathway for ATP [20,21], pannexin channels, specifically Panx1, have been shown to serve as potential exit pathways for ATP in stressed cells [22,23,24,25]. Alternatively, cell stress induced by exposing cells to pore-forming toxins, such as α-toxin, may result in alterations in mitochondrial integrity and reduction of ATP production, which may contribute to lowering cellular ATP content [26,27]. Thus, it is still unclear whether ATP production is attenuated in toxin-exposed cells or ATP gets lost from the cytosol to the external space through the Hla pore or other pathways that may be activated by signals elicited by attachment of Hla monomers or pore formation.

As airway epithelial cells generally use ATP release and purinergic signaling to control fluid secretion, mucus hydration and ciliary beat frequency [28] to adjust the rate of mucociliary clearance to momentary needs or to induce the secretion of interleukin-6 [29], we investigated the ability of cells to maintain ATP production and potential pathways of ATP release in immortalized human bronchial epithelial cells exposed to the major virulence factor of *Staphylococcus aureus*, alpha-toxin. 

## 2. Results

**Figure 1:**
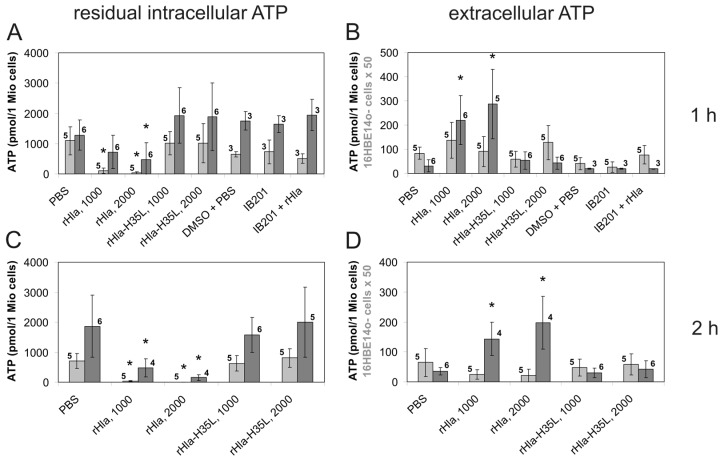
Residual intracellular ATP and extracellular ATP in airway epithelial cells exposed to *S. aureus* α-toxin (rHla), rHla-H35L or a blocker of the rHla-pore, IB201. The amounts of intracellular (**A**,**C**) or extracellular (**B**,**D**) ATP were determined using a luminometric assay in suspended 16HBE14o- cells (**light grey** bars) or S9 cells (**dark grey** bars) exposed for 1 h (**A**,**B**) or 2 h (**C**,**D**) to 1000 or 2000 ng/mL rHla or to the pore formation-deficient mutant rHla-H35L, or to the rHla pore blocker IB201 (1 µmol/L), respectively. Assays supplemented with phosphate-buffered saline (PBS) as a vehicle for rHla or dimethyl sulfoxide (DMSO) as a vehicle for IB201, respectively, served as controls. Data are presented as means ± S.D. (numbers of independent experiments as indicated by the numbers next to the bars). Testing the data series for acceptance of the H_0_ hypothesis (no differences of means of all data series) using ANOVA revealed that this hypothesis had to be declined (*p* > 0.05) for all series except for the data on extracellular ATP in 16HBE14o- cells treated for 2 h ((**D**), *p* < 0.05). Comparisons of individual means (experimental vs. PBS controls): * *p* < 0.05.

As shown in a previous study [17], airway epithelial cells that had been treated with recombinant *S. aureus* alpha-toxin (rHla) lost substantial amounts of cytosolic ATP. To elucidate the mechanism of ATP loss from rHla-exposed cells, we initially determined the basal values of ATP content in these cells by luminometric measurements of ATP contents in suspended cells. As shown in Figure 1A, the basal level of intracellular ATP content was approximately 1000 pmol/1 × 10^6^ cells in 16HBE14o- or in S9 cells. Assuming an average cell diameter of 12 µm for both cell types (estimates obtained from measuring freshly plated cell suspensions using a Luna Automated Cell Counter; Biozym, Hessisch Oldendorf, Germany), the intracellular ATP concentration was calculated to be 1.1 mmol/L in both cell types. Treatment of cells with *S. aureus* rHla (1000 or 2000 ng/mL) for 1 h lowered the intracellular ATP content by approximately 90% or 95% in 16HBE14o- cells or by approximately 45% or 60% in S9 cells, respectively (Figure 1A). The amounts of extracellular ATP increased under these conditions, but not as much as theoretically expected, considering the decrease in intracellular ATP. At 1 h of rHla-treatment (1000 ng/mL), the amount of extracellular ATP was approx. 3 pmol/1 × 10^6^ cells in 16HBE14o- cells or 370 pmol/1 × 10^6^ cells in S9 cells (Figure 1B). Under the assumption that ATP just leaves the cytoplasm to the external medium, these values represent just 0.3% of the expected level in 16HBE14o- cells or 41% of the expected level in S9 cells. After 2 h of rHla-exposure, the loss of ATP from the cytosol was even more pronounced (Figure 1C) and the accumulated amount of ATP in the extracellular medium was even smaller (Figure 1D) than that observed after 1 h, at least in 16HBE14o- cells (Figure 1B).

**Figure 2:**
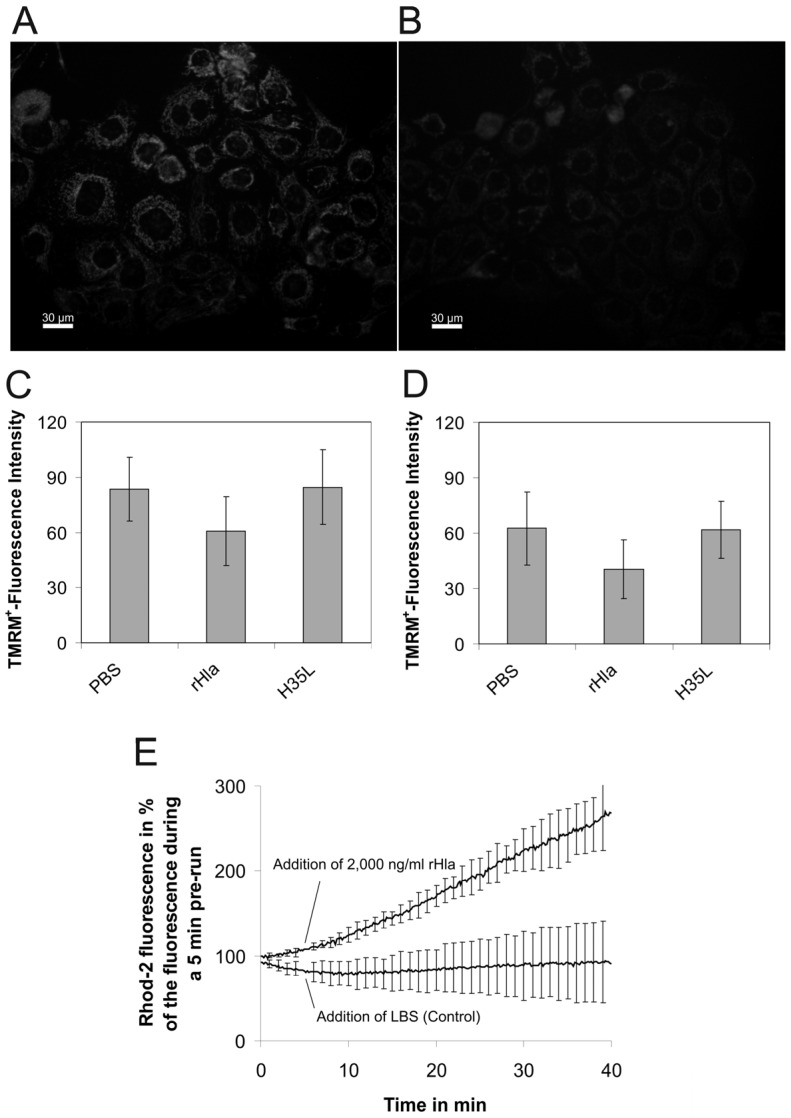
Exposure of airway epithelial cells to *S. aureus* α-toxin results in the reduction of inner mitochondrial membrane potential and calcium uptake into the matrix. 16HBE14o- or S9 cells were loaded for 1 h with 5 nmol/L of the fluorescent indicator dye tetramethylrhodamine methyl ester (TMRM^+^), which shows different subcellular localization and fluorescence intensities with changing values of the inner mitochondrial membrane potential. (**A**,**B**) examples of fluorescent images obtained in TMRM^+^-loaded 16HBE14o- cells kept under control conditions for 1 h (**A**) or after exposure to 2000 ng/mL rHla for 1 h (**B**); (**C**,**D**) TMRM^+^ fluorescence intensities in 16HBE14o- cells (**C**) or S9 cells (**D**) measured after 1 h of cell exposure to the vehicle (PBS, control), 2000 ng/mL rHla or rHla-H35L, respectively. Data are presented as means ± standard deviation (S.D.) (*n* = 4). Testing the data series for acceptance of the H_0_ hypothesis (no differences of means of all data series) using ANOVA revealed that this hypothesis had to be declined (*p* < 0.05) for 16HBE14o- cells (Figure 2C) and accepted for S9 cells (Figure 2D, *p* > 0.05). Comparisons of individual means (experimental vs. PBS controls) of data obtained using 16HBE14o- cells, however, did not reveal any significant differences; (**E**) Rhod 2-fluorescence indicating concentrations of free calcium ions in the mitochrondrial matrix of suspended and dye-loaded 16HBE14o- cells in the absence (control) or presence of 2000 ng/mL rHla (addition of vehicle or rHla at 5 min). Data are presented as means ± S.D. (*n* = 3).

As the portion of viable cells (trypan blue exclusion, LIVE/DEAD-assay) during the exposure period of 2 h to 2000 ng/mL rHla decreased at the most by 10% in both suspended as well as in adherent S9 or 16HBE14o- cells (results not shown), it was clear that the loss of intracellular ATP was not due to liberation of ATP from destroyed cells. Instead, cellular ATP production rate may have been attenuated or may have ceased altogether or, alternatively, ATP must have been released to the external medium through the plasma membranes of intact cells and metabolized in the extracellular space. To test the first hypothesis, we treated 80% confluent cultures of S9 or 16HBE14o- cells on 24-well plates with phosphate-buffered saline (PBS, control), 2000 ng/mL rHla or rHla-H35L for 1 h in the presence of 5 nmol/L tetramethylrhodamine methyl ester (TMRM^+^) as an indicator of changes in mitochondrial membrane potential. As shown in Figure 2, treatment of cells with rHla diminished mitochondrial membrane potential to approximately 60%–70% of the control level (PBS). Treatment of cells with an rHla-variant (rHla-H35L), which is not able to form functional transmembrane pores [30], did not affect mitochondrial membrane potential. As exposure of airway epithelial cells to rHla results in calcium influx into the cytosol [31] and mitochondria are able to buffer cellular calcium loading by importing calcium ions into the mitochondrial matrix [32], we measured [Ca^2+^] within the mitochondrial matrix using the fluorescent dye Rhod-2. As shown in Figure 2E, exposure of cells to 2000 ng/mL rHla resulted in an increase in [Ca^2+^]_m_ to approximately 250% of the initial value over a period of 40 min compared with the controls. These results are consistent with the hypothesis that mitochondrial ATP generation may have been somewhat attenuated in rHla-treated cells, but most likely did not cease entirely.

**Figure 3:**
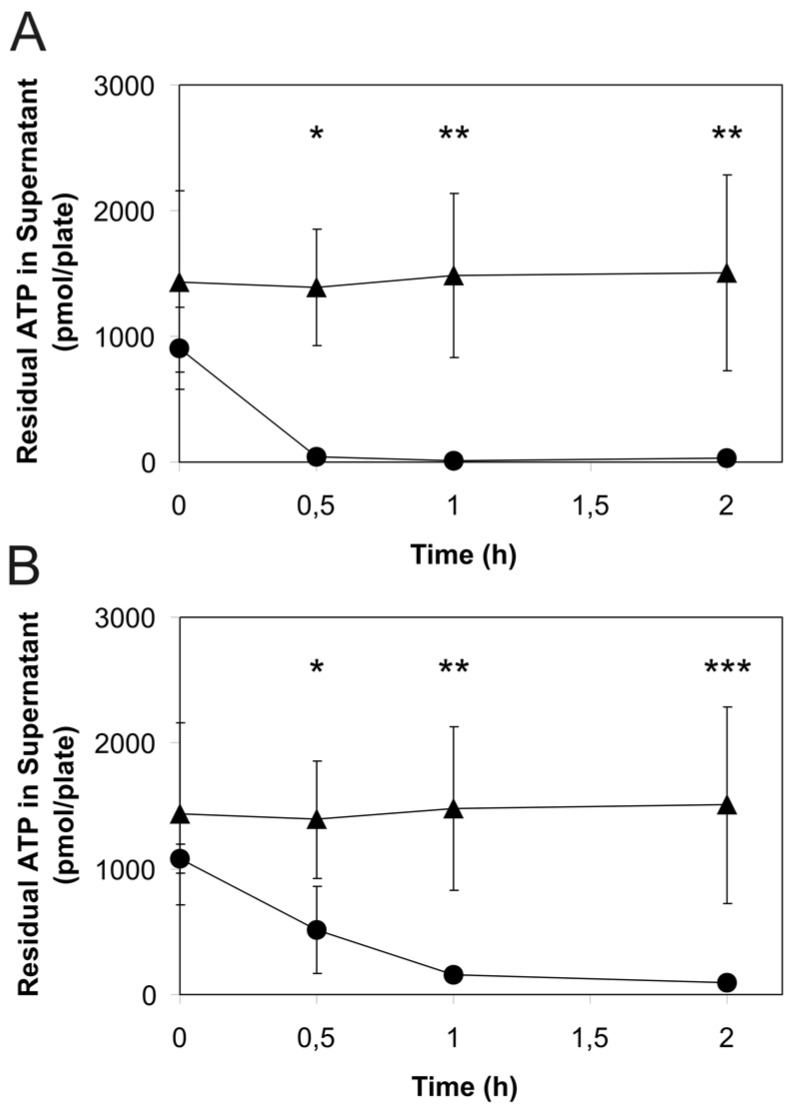
Degradation of ATP added to the extracellular medium of cultured 16HBE14o- or S9 airway epithelial cells. The amount of residual ATP was measured luminometrically in medium samples taken from confluent cultures of airway epithelial cells ((**A**) 16HBE14o-; (**B**) S9) after initial spiking of the medium with 0.3 µmol/L ATP. Results of assays performed in the presence of cells are indicated by dots, and those of assays performed in the absence of cells (controls) are indicated by triangles. Data are presented as means ± S.D. (*n* = 3). Significant differences of means compared with the controls: * *p* < 0.05, ** *p* < 0.01, *** *p* < 0.001.

To test the alternative hypothesis that cellular ATP is released to the external medium through the plasma membranes of intact cells and may be metabolized in the extracellular space, we monitored the rate extracellular ATP degradation in cultured intact cells whose culture medium had been spiked with ATP. As shown in Figure 3, the initial ATP concentration in the extracellular medium rapidly declined in the presence of cells, while it was stable over the entire experimental period when ATP was added to medium in cell culture plates without cells. Interestingly, the rate of ATP loss was higher in 16HBE14o- cells (Figure 3A) than that in S9 cells (Figure 3B), indicating that extracellular ATP degradation occurred more efficiently in 16HBE14o- cells.

To answer the question of whether ATP may be released through as yet unknown transmembrane channels activated by interaction of monomeric rHla molecules with cell surface receptors or by formation of the heptameric pre-pore on the outer surface of the cells, we incubated cells with 1000 or 2000 ng/mL rHla-H35L. As shown in Figure 1, there were no effects on the basal levels of intracellular ATP in cells that were exposed to rHla-H35L (1000 or 2000 ng/mL) for 1 or 2 h (Figure 1A,C), and there was no indication that any ATP accumulated in the extracellular space (Figure 1B,D). This demonstrates that formation of functional rHla-pores was required to allow ATP to exit the cells.

**Figure 4:**
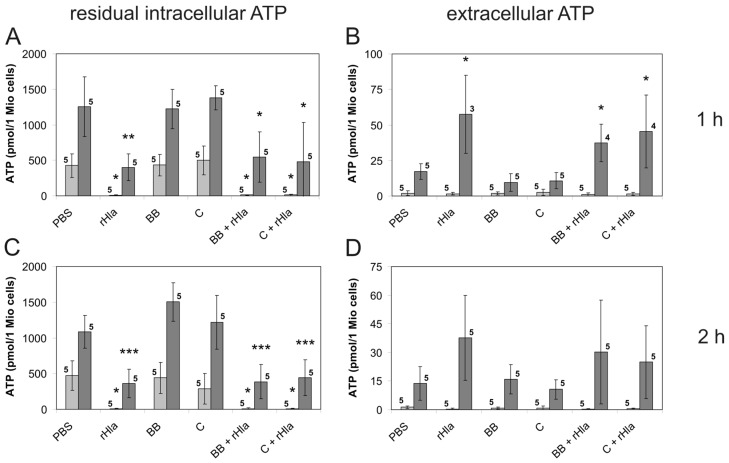
Residual intracellular ATP and extracellular ATP in airway epithelial cells exposed to *S. aureus* rHla in the absence or in the presence of Panx1 channel blockers. The amounts of intracellular (**A**,**C**) or extracellular (**B**,**D**) ATP were determined using a luminometric assay in suspended 16HBE14o- cells (**light grey** bars) or S9 cells (**dark grey** bars) exposed for 1 h (**A**,**B**) or 2 h (**C**,**D**) to 2000 ng/mL rHla or to the Panx1 pore blockers brilliant blue FCF (5 µmol/L) or carbenoxolone (10 µmol/L), respectively. Assays supplemented with PBS as the vehicle for these agents served as controls. Data are presented as means ± S.D. (numbers of independent experiments as indicated by the numbers next to the bars). Testing the data series for acceptance of the H_0_ hypothesis (no differences of means of all data series) using ANOVA revealed that this hypothesis had to be declined (*p* > 0.05) for all series except for the data on extracellular ATP in cells (16HBE14o- as well as S9) treated for 2 h ((**D**) *p* < 0.05). Comparisons of individual means (experimental vs. PBS controls): * *p* < 0.05, ** *p* < 0.01, *** *p* < 0.001.

To test the hypothesis that ATP leaves the cells through transmembrane pores formed by *S. aureus* Hla, we exposed cells to rHla in the presence of 1 µmol/L of the cyclodextrin-derivative IB201, which has been shown to diffuse into intact cells [33,34] and to block the permeability of the Hla pore by inserting itself into the pore from the intracellular side [35]. Pre-treatment of cells With 1 µmol/L IB201 entirely suppressed the ATP-releasing effect of rHla (Figure 1A) and the accumulation of ATP in the extracellular medium (Figure 1B). As nothing is known about the potential blocking effects of IB201 on pannexin-1 channels, which may be potential alternative exit pathways for ATP for airway epithelial cells [25], we pre-incubated our cells for 10 min with blockers of pannexin-1 channels, either 5 µmol/L brilliant blue FCF [36] or 10 µmol/L carbenoxolone [37] before adding rHla to the cells. While each of these inhibitors alone did not affect the basal level of intra- or extracellular ATP in either cell type (Figure 4A,B), subsequent addition of 2000 ng/mL rHla resulted in ATP loss from the cytosol (Figure 4A,C), which was not different from that elicited by rHla without the inhibitors (Figure 1A). The rHla-mediated loss of intracellular ATP was accompanied by moderate increases in the amount of extracellular ATP in S9-, but not in 16HBE14o- cells (Figure 4B,D).

## 3. Discussion

Many cells under mechanical or chemical stress release cytosolic adenosine triphosphate (ATP) to the extracellular space. This may occur via vesicle exocytosis [38] or through endogenous transmembrane channels whose subunits have been only recently identified as members of the pannexin family [23,24,39]. Specifically, pannexin 1 (Panx1) has been implicated in physiological ATP release from the cytosol, especially in epithelial cells [25,40]. Extracellular ATP may be utilized as a danger signal originating from stressed cells and inducing other cells to respond properly to the stress situation either by activation of defensive mechanisms or by inducing cell death [41,42]. In airway epithelial cells, extracellular ATP may bind to and activate purinergic receptors localized in the apical plasma membrane [18,43]. ATP-mediated ionotropic (P2X receptors) or metabotropic (P2Y receptors) signaling may occur in an autocrine or a paracrine fashion and result in changes in ciliary beat frequency in ciliated cells [44], fluid secretion in salt and water transporting cells [45], and mucus secretion in goblet cells [46,47], or may induce activation of the innate immune system by mediating release of interleukin-6 [29]. These effects may be terminated by degradation of extracellular ATP through dephosphorylation mediated by extracellular ATPases (ectonucleotide-phosphodiesterase/pyrophosphatase [48]) or elongated by ecto-adenylate kinases regenerating extracellular adenosine diphosphate (ADP) or ATP [48,49]. Extracellular adenosine monophosphate (AMP) may be further converted by the ecto-5′-nucleotidase to adenosine, which signals through P1 adenosine receptors to a variety of different cell types [50] including airway epithelial cells, in which it activates fluid secretion via accumulation of cyclic adenosine monophosphate (cAMP) in the cytosol [51].

In this study, we were able to confirm previous observations [17] that exposure of human immortalized airway epithelial cells to *Staphylococcus aureus* alpha-toxin (Hla) resulted in loss of cytosolic ATP and some accumulation of extracellular ATP (Figure 1). However, extracellular ATP did not accumulate proportionally to the amounts of ATP released from the cells. There are two possible explanations for this finding. The first is that rHla pore formation which has been shown to result in an influx of sodium and calcium ions into the cytosol, and plasma membrane depolarization [31] reduces mitochondrial inner membrane potential and mitochondrial ATP production. This would lower the cellular ATP content per se without any effects on extracellular ATP. Measurements of changes in intra-mitochondrial calcium concentration ([Ca^2+^]_m_) seemed to confirm that calcium ions enter the mitochondrial matrix and elevate the concentration of free calcium ions (Figure 2E). However, increases in [Ca^2+^]_m_ in the lower micromolar range have been shown to increase ATP generation by positively modulating the activity of Krebs cycle enzymes [52]. On the other hand, it has also been found that rapid calcium uptake by mitochondria can indeed attenuate ATP production by lowering the mitochondrial membrane potential [53]. In our experiments, the increase in [Ca^2+^]_m_ was accompanied by a reduction in mitochondrial membrane potential (Figure 2A–D). It may well be that these two observations are causally related as influx of positive charges (Ca^2+^ ions) into the matrix may lower the potential difference between matrix and cytosol. However, it is well known that calcium uptake into the mitochondrial matrix does not necessarily result in large elevations in [Ca^2+^]_m_ (and subsequent changes in mitochondrial membrane potential) because large quantities of excess calcium ions may be precipitated with phosphate ions within the matrix [52]. This effect, in turn, may also protect the mitochondrial membrane potential. In our experiments, the mitochondrial membrane potential remained at 60%–70% of the initial level, and, additionally, there was some degree of ATP accumulation in the extracellular space in rHla-treated cells (Figure 1B) that would probably have not occurred if the cellular ATP pool would be fully depleted by lack of mitochondrial ATP synthesis. Thus, it seems reasonable to assume that a combination of effects was responsible for the observed effects of rHla-mediated losses of ATP including a reduction in the rate of mitochondrial ATP regeneration associated with a decline in cellular ATP content caused by an rHla-induced ATP-efflux through the plasma membrane followed by extracellular degradation of ATP by ecto-enzymes. Such enzyme activities may be more abundant on the extracellular surface of 16HBE14o- cells compared with S9 cells as the degradation of ATP in spiked cell culture medium occurred more rapidly in 16HBE14o- than in S9 cells (Figure 3). Unfortunately, we were not able to consistently analyze ATP in the presence of ectonucleotidase inhibitors (polyoxometalate 1 or α,β-methylene adenosine 5′-diphosphate, respectively) for technical reasons, so that we cannot clearly quantify the relative contribution of each of these mechanisms to the overall effects.

As the ATP release from 16HBE14o- or S9 cells occurred rapidly (Figure 1A), it is most likely that ATP is released through transmembrane channels permeable to ATP. As such channels may be activated by interaction of monomeric or polymeric rHla molecules with receptor sites at the cell surface [7,54,55], we used a mutant of Hla, rHla-H35L, that mimics attachment of Hla to the cell surface, oligomerization and formation of a pre-pore, but does not form a functional transmembrane pore [30], in order to test whether it was able to mediate ATP release. Application of this mutant did not change the distribution of ATP between intra- and extracellular space of airway epithelial cells (Figure 1), indicating that formation of functional rHla pores was required for ATP release from these cells.

The open question of whether ATP leaves the cells through endogenous transmembrane channels activated by exposure of cells to rHla or directly through the transmembrane pores formed by heptamers of rHla molecules was addressed using a blocker of the functional Hla pore IB201 [34]. IB201 completely abolished rHla-mediated ATP release from the experimental cells (Figure 1A). There was also no indication of extracellular ATP accumulation (Figure 1B), indicating that at least a fraction of cytosolic ATP leaves the cells directly through the transmembrane pores formed by rHla heptamers.

As we do not know anything about the potential sensitivity of the Panx1 channels to IB201 and could, therefore, not exclude the possibility that some ATP may have left the rHla-treated cells via Panx1 channels, we measured rHla-mediated ATP release in the presence of Panx1 channel blockers, carbenoxolone [37] or brilliant blue FCF [36]. We used two Panx1-inhibitors that should have different inhibitory mechanisms, in order to make sure that at least one of them specifically attenuates potential Panx1-mediated ATP release because there is no specific stimulus known to activate pannexin channels in cultured airway epithelial cells. As shown in Figure 4, none of these inhibitors had effects on the basal levels of ATP in intra- or extracellular spaces and did not interfere with rHla-mediated ATP release. This shows that Panx1 channels are most likely not involved in the rHla-induced ATP permeability of the plasma membrane in these cells.

Taken together, alpha-toxin mediated the release of ATP from bronchial epithelial cells. This response requires formation of functional transmembrane pores. It seems likely that ATP release occurs directly through the pore formed by the toxin and not via endogenous channels (e.g., Panx1 channels). Extracellular ATP is metabolically processed, most likely by ecto-enzymes, a reaction that seems to occur with higher efficiency in 16HBE14o- cells compared with S9 cells. Whether such a difference has any implications for the observed differences in sensitivities against alpha-toxins in these cell types [4] remains to be clarified.

## 4. Materials and Methods

### 4.1. Chemicals and Reagents

The mitochondrial membrane potential probe TMRM^+^ and the ATP Determination Kit were obtained from Life Technologies (Darmstadt, Germany). The calcium indicator dye Rhod-2 AM was purchased from Invitrogen, Paisley, UK. The disodium salt of carbenoxolone and brilliant blue FCF were obtained from Sigma-Aldrich (Munich, Germany). The β-cyclodextrin derivative IB201 (ANBOβCD) was obtained from BEI Resources (Manassas, VA, USA). Trypsin (including ethylenediaminetetraacetic acid (EDTA)) and cell culture medium (Eagle’s Minimal Essential Medium) were purchased from GE-Healthcare (Freiburg, Germany). Fetal bovine serum (Superior) and penicillin/streptomycin were obtained from Biochrom (Berlin, Germany). All other chemicals were reagent grade and obtained from Roth (Karlsruhe, Germany).

### 4.2. Cell Culture

Immortalized human airway epithelial cells (16HBE14o- or S9 cells) were cultured on 10 cm Cell+ plates (Sarstedt, Numbrecht, Germany) at 37 °C and gassing with 5% CO_2_. Eagle’s MEM (PAN Biotech, Aidenbach, Germany) containing 10% FBS superior (Biochrom, Berlin, Germany), and 1% (*w*/*v*) penicillin/streptomycin (PAA Laboratories, Cölbe, Germany) was routinely used as cell culture medium and changed every 3–4 days. Shortly before cells formed confluent monolayers, they were passaged (1:10) or directly used in the experiments.

Expression and purification of recombinant *Staphylococcus aureus* alpha-toxin.

Recombinant alpha-toxin (hemolysin A, rHla) was prepared as described previously [12,56]. The purity of rHla was assessed by SDS-PAGE (sodium dodecyl sulfate-polyacrylamide gel electrophoresis) after staining the gels with Coomassie brilliant blue. Biological activity of every batch of recombinant Hla was checked using a β-hemolysis assay on blood agar plates (Columbia agar with 5% sheep blood; Becton Dickinson, Heidelberg, Germany). The concentrations of rHla routinely used were 1000 or 2000 ng/mL (30 or 60 nmol/L) for reasons discussed previously [57].

For construction of an H35L mutant of hemolysin A, which does not form functional pores in the plasma membranes of eukaryotic host cells [30] and is used as a negative control for rHla, we changed the wildtype sequence CACAAA (encoding amino acid residues H35 and K36) into CTTAAG (encoding amino acid residues L35 and K36), thereby generating a recognition site for the restriction enzyme *Bsp*T1. Overlapping and reverse orientated primers (forward: 5′-TTCTTAAGCATGCCATTTTCTTTATCATAAG-3′; reverse: 5′-TTCTTAAGCATGCCATTTTCTTTATCATAAG-3′) were derived from the pPR-IBA1 expression vector (IBA, Göttingen, Germany) containing the wildtype *hla*-sequence. The plasmid was amplified in a PCR reaction using a T-gradient thermocycler (Biometra, Goettingen, Germany) using PhusionTM High-Fidelity DNA polymerase (Thermo Scientific, Schwerte, Germany). The amplification reaction was separated on a 1% agarose gel, and a gel slice containing the amplificate (about 3.7 kb in size) was cut out and DNA was purified using the Silica Bead DNA Gel Extraction Kit (Thermo Scientific, Schwerte, Germany). The purified amplificate was subsequently digested with *Bsp*T1 (thus generating sticky *Bsp*T1-ends), the reaction was separated on a 1% agarose gel, and the DNA isolated and purified. The fragment was then circularized using T4 DNA Ligase (Thermo Scientific, Schwerte, Germany) and transformed into *E. coli* DH5α. Plasmid DNA of pPR-Hla-H35L was purified and the complete coding sequence of hemolysin A-H35L was determined. Recombinant Hla-H35L was prepared, tested and used as described above for recombinant Hla.

### 4.3. Mitochondrial Inner Membrane Potential

16HBE14o- or S9 cells grown to 80% confluency in 24-well cell culture plates (83.1836.300, Sarstedt, Newton, NC, USA) were loaded for 1 h with 5 nmol/L of the fluorescent indicator dye TMRM^+^, which diffusively partitions into the cytosol (due to its negative potential with respect to the extracellular space) and into the matrix of the mitochondria (due to the negative potential of the mitochondrial matrix with respect to the cytosol). The fluorescence intensity of the dye changes with its subcellular localization and concentration, and is, therefore, a good measure for changes in mitochondrial membrane potential [58]. Images of the cell layers were taken using an ECLIPSE TE300 fluorescence (filter block G2A) microscope (Nikon, Düsseldorf, Germany) equipped with a DXM 1200 digital camera (Nikon, Düsseldorf, Germany). The mean of the fluorescence intensity of the ten squares (2 × 2 µm) of the mitochondrial network areas in each image with the brightest fluorescence was taken as a proxy for mitochondrial inner membrane potential.

### 4.4. Calcium Concentration in the Mitochondrial Matrix

Suspended 16HBE14o- cells were loaded for 20 min with 5 µmol/L Rhod 2-AM (Enzo, Farmingdale, NY, USA) at 37 °C in the dark. Cells were washed and re-suspended in 4-(2-hydroxyethyl)-1-piperazineethanesulfonic acid (HEPES)-buffered low bicarbonate buffer solution (LBS) containing (in mmol/L) 132.5 NaCl, 4.8 KCl, 1.2 MgSO_4_, 1.2 KH_2_PO_4_, 1.3 CaCl_2_, 6.0 glucose and 15.0 HEPES, pH 7.4 [59] and allowed to recover for another 20 min. During this time, residual intracellular Rhod 2-AM was cleaved. Cells were suspended in LBS in the cuvette of a spectrofluorimeter (FluoroMax-3, HORIBA Jobin Yvon, Bensheim, Germany), and fluorescence was monitored at excitation and emission wavelengths of 550 nm or 580 nm, respectively. 

### 4.5. Sample Preparation for Assaying Intra- and Extracellular ATP Concentrations

Cultured cells on 10 cm-plates were washed using 5 mL PBS containing (in mmol/L) 137 NaCl, 2.7 KCl, 10.1 Na_2_HPO_4_·2H_2_O, 1.8 KH_2_PO_4_, pH 7.4, briefly trypsinized, washed by centrifugation (2 min at 600× *g*) and re-suspended in 1 mL LBS. Cells of one plate were re-suspended in 500 µL LBS. An aliquot of this suspension was diluted and used to count the number of cells using a Neubauer chamber. Cells were treated with 1000 or 2000 ng/mL rHla or rHla-H35L, respectively, or phosphate-buffered saline (PBS, control) in the absence or presence of either 5 µmol/L brilliant blue FCF, 10 µmol/L carbenoxolon (10 min pre-incubation, each), or 1 µmol/L IB201 (20 min pre-incubation), respectively, for 1 or 2 h at 37 °C. After the incubation period, cells were spun down (600× *g*, 2 min) and the supernatant was collected, aliquotted (4 × 100 µL) and frozen at −80 °C. The cell pellet was re-suspended in 500 µL hypotonic buffer solution (10 mmol/L triethanolamine, 5 mmol/L EDTA, 0.5 mmol/L Pefablock, 10 μg/mL trypsin inhibitor, pH 6.8), and rapidly frozen in liquid nitrogen and thawed twice. Membrane debris was spun down (13,000× *g*, 3 min, 4 °C) and the supernatant containing the residual intracellular ATP was collected, aliquotted (4 × 100 µL) and frozen at −80 °C. In parallel to these experiments, cell viability was tested under the different assay conditions. Viability tests (trypan blue exclusion) were performed by mixing equal volumes of cell suspension and 0.4% trypan blue staining solution (Logos Biosystems, Anyang, South Korea) and immediately counting in a Neubauer cell counting chamber. Equivalent assays of cell viability were performed in adherent cells using the LIVE/DEAD Fixable Green Dead Cell Stain (ThermoFisher Scientific, Darmstadt, Germany) according to the manufacturer’s instructions. Images of the cell layers were generated using an ECLIPSE TE300 fluorescence microscope equipped with a DXM 1200 digital camera (Nikon, Düsseldorf, Germany). The number of green fluorescent (dead) cells were counted and expressed as percentage of the total cell number per image.

### 4.6. Estimation of the Rates of Extracellular ATP Degradation

Cultured cells on 10 cm plates were grown to confluency, washed using pre-warmed 5 mL PBS and covered with 5 mL HEPES-buffered saline. The experiment was started by adding 0.3 µmol/L ATP. Upon mixing by swirling the culture plate, cells were incubated for 2 h at 37 °C. Samples (2 × 100 µL) were taken at 0, 0.5, 1 and 2 h and rapidly frozen at −80 °C. The same procedure was conducted using cell culture plates without cells to control for background ATP degradation.

### 4.7. Luminometric ATP Assay

ATP concentrations in the samples were luminometrically determined using the ATP Determination Kit from Life Technologies (Darmstadt, Germany) and an Infinite M200Pro microplate reader (Tecan, Crailsheim, Germany) equipped with the software package I-control V1.11 (Tecan, Grödig, Austria). The assays were conducted according to the protocol provided by the kit manufacturer. Standard curves were prepared using solutions with known ATP concentrations in the relevant range. The results were expressed as amounts of ATP (in pmol per 1 × 10^6^ cells).

### 4.8. Data Presentation and Statistics

Means (±S.D.) were calculated from double determinations of ATP amounts from experiments on different cell preparations. As the results for each of the treatments were statistically tested against the control data, we performed ANOVA on each of the data series. Individual means (experimental/control) were tested for significant differences using Student’s *t*-test (for comparisons of means of equal variances) or the Wilcoxon-Mann-Whitney test (for comparison of means of different variances). Significant differences of means were assumed with *p* < 0.05.
